# Anxiety and Depressive Symptoms Among Early‐Year Medical Students in Pakistan: Prevalence, Associated Factors, and Mental Health Service Implications—A Multicenter Cross‐Sectional Study

**DOI:** 10.1002/hsr2.73250

**Published:** 2026-09-19

**Authors:** Farah Rashid, Iffat Noor, Wajeha Najeeb, Hadiya Rashed Siddiqui, Hafiz Muhammad Ali, Abid Malik, Atif Rahman

**Affiliations:** ^1^ Department of Community Medicine/Public Health, NUST School of Health Sciences National University of Sciences and Technology (NUST) Islamabad Pakistan; ^2^ Health Services Academy Islamabad Pakistan; ^3^ Department of Community Medicine/Public Health CMH Kharian Medical College Kharian Pakistan; ^4^ Islamabad Medical & Dental College Islamabad Pakistan; ^5^ Department of Public Mental Health Health Services Academy Islamabad Pakistan; ^6^ Department of Primary Care and Mental Health, Institute of Population Health University of Liverpool Liverpool UK

**Keywords:** anxiety, depressive symptoms, medical students, mental health, Pakistan

## Abstract

**Background:**

Anxiety and depressive symptoms are common among medical students, but multicenter evidence from the early years of training in Pakistan is limited. We estimated symptom prevalence and examined sociodemographic, behavioral, academic, and psychosocial correlates of symptom severity.

**Methods:**

We conducted a multicenter cross‐sectional survey from January to June 2024 among 1630 of 1800 invited first‐ and second‐year students at 9 medical colleges in Islamabad (response rate, 90.6%). Symptoms were assessed with the Patient Health Questionnaire‐9 (PHQ‐9) and Generalized Anxiety Disorder‐7 (GAD‐7). Because the proportional‐odds assumptions were not supported and sparse upper‐severity categories caused separation, adjusted associations were estimated using bias‐reduced multinomial logistic regression with minimal symptoms as the reference outcome.

**Results:**

Anxiety symptoms (GAD‐7 ≥ 5) were present in 756/1630 students (46.4%; 95% CI, 44.0%–48.8%), and depressive symptoms (PHQ‐9 ≥ 5) in 942/1630 (57.8%; 95% CI, 55.4%–60.2%). Peer pressure was associated with moderate versus minimal anxiety (aOR, 6.42; 95% CI, 3.59–11.49), severe versus minimal anxiety (aOR, 37.23; 95% CI, 3.54–391.86), moderate versus minimal depressive symptoms (aOR, 4.07; 95% CI, 2.69–6.18), and moderately severe/severe versus minimal depressive symptoms (aOR, 10.79; 95% CI, 3.79–30.73). Sleeping 7–8 h per night was associated with lower odds of severe anxiety (aOR, 0.11; 95% CI, 0.02–0.59) and moderate depressive symptoms (aOR, 0.22; 95% CI, 0.12–0.38). Severe‐category estimates were imprecise because only 20 students had severe anxiety and 34 had moderately severe/severe depressive symptoms.

**Conclusion:**

Anxiety and depressive symptoms were common among early‐year medical students. Peer pressure, harassment, sleep, academic stage, and support were associated with symptom severity, but the cross‐sectional design precludes causal inference. Medical colleges should evaluate confidential student‐support and referral pathways.

## 1 Introduction

Mental health symptoms among university students are a substantial public health and educational concern. Medical training combines a demanding workload, performance pressure, and emotionally challenging experiences, while stigma and uncertainty about confidentiality may discourage help‐seeking [1, 2].

Meta‐analyses have estimated pooled prevalences of 27.2% for depression or depressive symptoms and 33.8% for anxiety among medical students, although estimates vary by setting, instrument, cutoff, sampling strategy, and educational stage [3, 4]. These are screening estimates and should not be interpreted as confirmed psychiatric diagnoses. Previous studies have described correlates of psychological distress among medical students, including academic pressure, transition into medical training, sleep disruption, limited physical activity, interpersonal stress, harassment, weak social support, and maladaptive coping strategies [5, 6, 7, 8]. Examining these domains concurrently may help distinguish broad patterns that warrant further longitudinal study and institutional attention.

Psychological distress during training may coexist with impaired academic functioning, suicidal ideation, reduced well‐being, and unmet support needs [9, 10]. In Pakistan, constraints in mental‐health policy implementation, service availability, workforce, and integration with primary care may further limit access to appropriate support [11]. The present survey did not assess psychiatric diagnosis, treatment uptake, service utilization, educational interruption, or economic consequences.

Financial and health‐system implications are therefore considered only as contextual hypothesis. The study does not estimate treatment costs, family expenditure, service demand, or cost‐effectiveness. Any recommendations are framed as service‐planning considerations that require independent evaluation rather than as interventions proven effective by this cross‐sectional study.

This study was informed by the promotion of mental health and well‐being within Sustainable Development Goal 3 and by calls for accessible, rights‐based mental‐health services [12, 13]. Its objectives were to: (1) estimate the prevalence of anxiety and depressive symptoms among first‐ and second‐year medical students at participating colleges in Islamabad; (2) examine sociodemographic, behavioral, academic, and psychosocial factors associated with symptom severity; and (3) consider implications for student‐support planning without inferring causality or unmeasured financial effects.

### 1.1 Conceptual Framework

**Figure 1:**
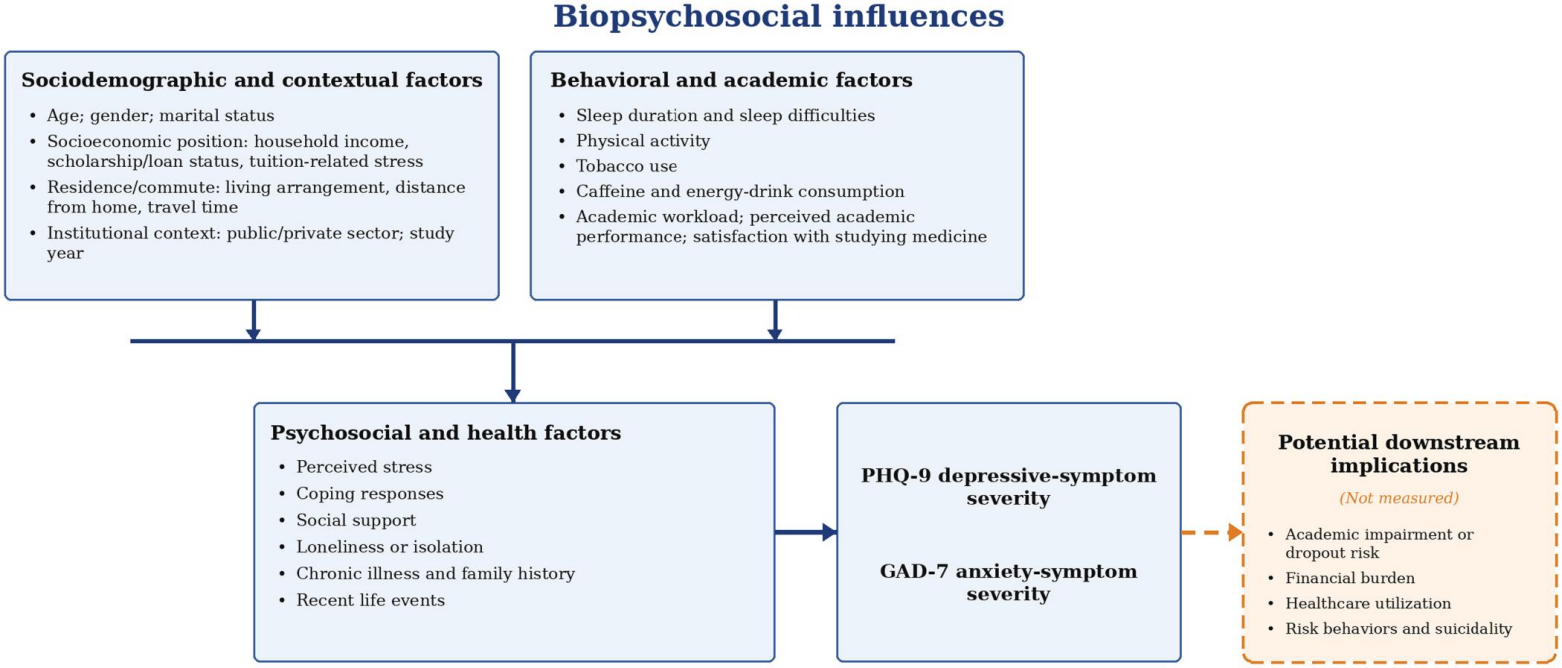
Conceptual framework for depressive‐ and anxiety‐symptom severity among first‐ and second‐year medical students. Solid arrows indicate hypothesized cross‐sectional relationships among measured biopsychosocial factors and PHQ‐9/GAD‐7 symptom severity; they do not imply causal or mediating pathways. The dashed arrow indicates potential downstream implications that were not measured. PHQ‐9 and GAD‐7 are screening instruments and do not establish clinical diagnoses.

The conceptual framework, as shown in Figure 1, integrates the biopsychosocial model and the transactional stress‐and‐coping model [14, 15]. It proposes that sociodemographic, behavioral, academic, and psychosocial characteristics may be associated with PHQ‐9 and GAD‐7 symptom severity through stress appraisal, coping responses, and available support. The framework distinguishes measured predictors and outcomes from downstream educational, financial, and health‐service implications, which were not measured.

## 2 Materials and Methods

### 2.1 Study Design and Setting

This multicenter cross‐sectional study was conducted from January to June 2024 at nine medical colleges in Islamabad, Pakistan. Reporting was guided by the Strengthening the Reporting of Observational Studies in Epidemiology (STROBE) statement [16].

### 2.2 Participants and Sampling

Eligible participants were undergraduate medical students enrolled in the first or second academic year, aged 17 years or older, and able to provide written informed consent. Students who reported current psychiatric treatment were excluded under the original protocol, and questionnaires with more than 20% missing responses were excluded. Colleges were selected according to accessibility and institutional permission. Within participating colleges, students were stratified by academic year and all eligible students in the selected strata were invited. Of 1800 students recorded as invited or eligible, 1630 participated (response rate, 90.6%). College identifiers were removed from the deidentified analytical data set to protect institutional confidentiality. Consequently, college‐level clustering could not be evaluated or incorporated into the regression models. If responses were correlated within colleges, the reported standard errors and confidence intervals may be underestimated.

### 2.3 Data Collection

Data were collected using an on‐site, structured, self‐administered questionnaire during scheduled class time. Participants received information about the study purpose, voluntary participation, privacy, and anonymity. Trained research assistants supervised data collection.

### 2.4 Outcome Measures

Depressive‐symptom severity was measured with the 9‐item Patient Health Questionnaire (PHQ‐9), which assesses symptoms during the preceding 2 weeks (score range, 0–27). Descriptive categories were minimal (0–4), mild (5–9), moderate (10–14), moderately severe (15–19), and severe (20–27) [17].

Anxiety‐symptom severity was assessed with the 7‐item Generalized Anxiety Disorder scale (GAD‐7; score range, 0–21), categorized as minimal (0–4), mild (5–9), moderate (10–14), and severe (15–21) [18]. Both instruments are screening measures and do not establish psychiatric diagnoses.

### 2.5 Predictor Measures

The predictors reported in the study are: gender, residential status, academic year, co‐curricular participation, physical activity level, sleep pattern, family history of mental illness, harassment history, peer pressure, access to counseling services, strong social support, family support, coping mechanism, screen time, and academic performance. All covariates were entered simultaneously.

Except for the PHQ‐9 and GAD‐7, the categorical predictors were study‐specific self‐report items rather than validated multidimensional instruments. Peer pressure referred to perceived pressure or influence from classmates on academic, social, or personal behavior. Harassment history referred to self‐reported exposure to unwanted, intimidating, humiliating, or distressing behavior in an educational or social setting. Coping mechanism was classified as positive or negative according to the participant's principal reported response to stress. Family and social support reflected the perceived availability of support. These broad indicators did not quantify frequency, duration, severity, source, or context and should not be interpreted as equivalent to validated scales.

### 2.6 Statistical Analysis

Categorical variables were summarized as *n*/*N* (%). Age was available only in prespecified categories and was therefore not summarized as a mean and standard deviation. Screening prevalence was defined as PHQ‐9 ≥ 5 for depressive symptoms and GAD‐7 ≥ 5 for anxiety symptoms; Wilson 95% confidence intervals were calculated. The same 1630 complete observations were available for all variables in the final harmonized models, so complete‐case analysis did not exclude any participant. Because only five students reported failing academic performance, failing and poor performance were combined for multivariable modeling. Multicollinearity was assessed from the model design matrix; the maximum variance inflation factor was 2.87.

Both outcomes were initially considered as ordered categories. The parallel‐slopes assumption was not supported for the final depression or anxiety model, and a single common proportional odds ratio was therefore not reported. Ordinary maximum‐likelihood multinomial models also showed complete or quasi‐complete separation in sparse upper‐severity categories. Consequently, bias‐reduced multinomial logistic regression using Jeffreys‐prior penalization was fitted for both outcomes [19]. Minimal symptoms were the reference outcome. For depression, moderately severe and severe categories were combined for multivariable modeling because only eight students were in the severe category; all five categories were retained in descriptive reporting. Separate adjusted odds ratios (aORs) and 95% confidence intervals are reported for each outcome contrast.

All tests were two‐sided with *α* = 0.05; interpretation emphasizes effect size and precision rather than statistical significance alone [20, 21]. The analysis was conducted by IBM SPSS Statistics 31.0 format.

## 3 Results

### 3.1 Participant Characteristics

All 1630 participants had complete data for the final model variables. The recorded age categories were < 18 years for 28/1630 (1.7%), 18–20 years for 1273/1630 (78.1%), 21–23 years for 315/1630 (19.3%), and 24–26 years for 14/1630 (0.9%). The sample included 1100 women (67.5%) and 530 men (32.5%); 797 students (48.9%) were in the first year and 833 (51.1%) in the second year. A total of 723 (44.4%) lived in hostels and 907 (55.6%) with family. Nuclear‐family structure was reported by 1219 (74.8%) and joint‐family structure by 411 (25.2%). Socioeconomic status was reported as low income by 57 (3.5%), middle income by 1302 (79.9%), and high income by 271 (16.6%).

### 3.2 Prevalence and Severity Distributions

**Figure 2:**
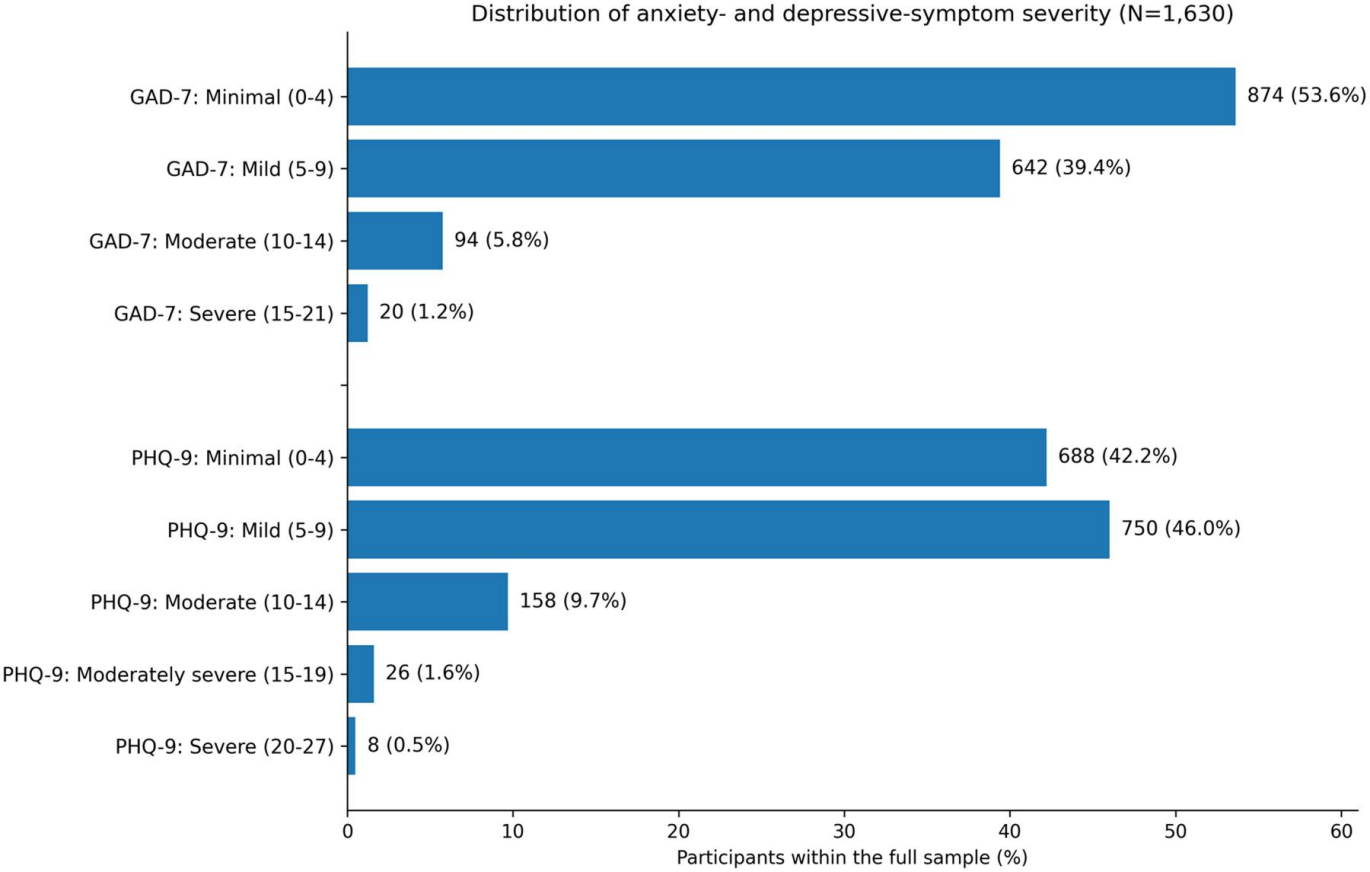
Distribution of GAD‐7 anxiety‐symptom and PHQ‐9 depressive‐symptom severity among first‐ and second‐year medical students (*N* = 1630). Bars show the percentage of the full sample; labels give *n* (%). GAD‐7 categories were minimal (0–4), mild (5–9), moderate (10–14), and severe (15–21). PHQ‐9 categories were minimal (0–4), mild (5–9), moderate (10–14), moderately severe (15–19), and severe (20–27).

**Figure 3:**
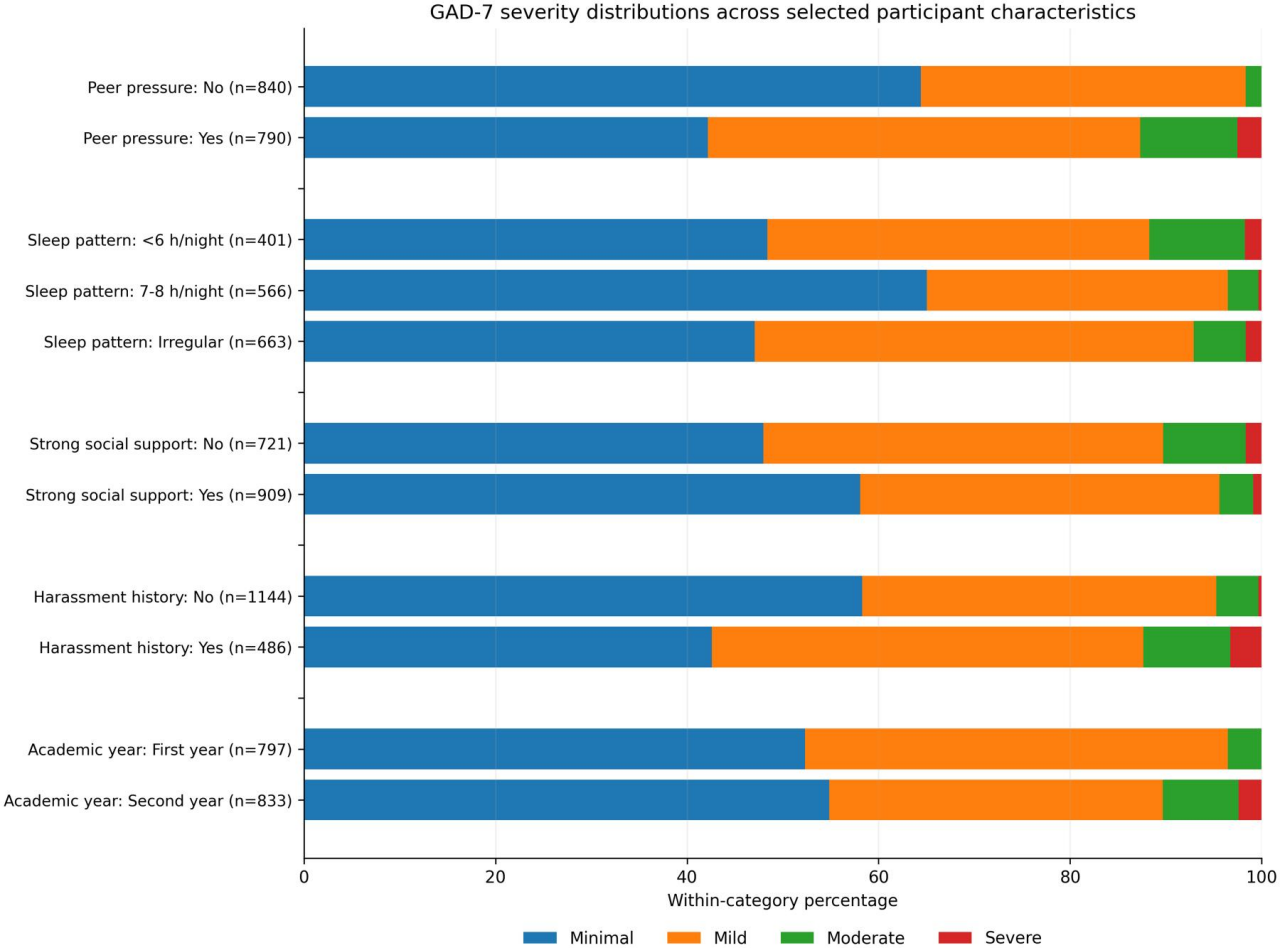
Distribution of GAD‐7 anxiety‐symptom severity across selected participant characteristics (*N* = 1630). Each horizontal bar shows percentages within the named characteristic category; category‐specific denominators are given in the labels. These are descriptive cross‐tabulations and do not establish independent, temporal, or causal associations.

**Figure 4:**
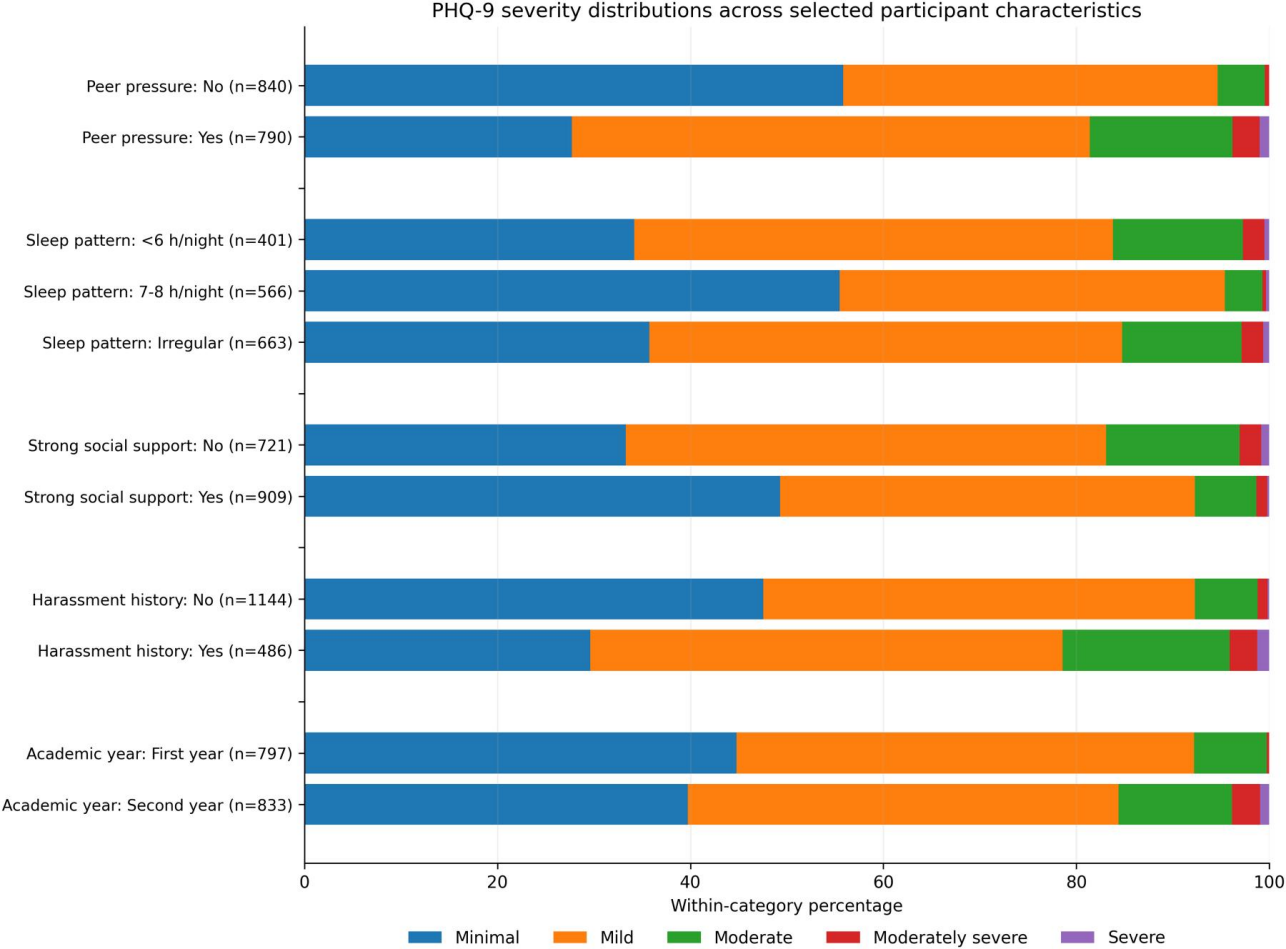
Distribution of PHQ‐9 depressive‐symptom severity across selected participant characteristics (*N* = 1630). Each horizontal bar shows percentages within the named characteristic category; category‐specific denominators are given in the labels. These are descriptive cross‐tabulations and do not establish independent, temporal, or causal associations.

Anxiety symptoms at or above the mild threshold (GAD‐7 ≥ 5) were present in 756/1630 participants (46.4%; 95% CI, 44.0%–48.8%). The GAD‐7 categories were minimal in 874 (53.6%), mild in 642 (39.4%), moderate in 94 (5.8%), and severe in 20 (1.2%). Depressive symptoms at or above the mild threshold (PHQ‐9 ≥ 5) were present in 942/1630 participants (57.8%; 95% CI, 55.4%–60.2%). The PHQ‐9 categories were minimal in 688 (42.2%), mild in 750 (46.0%), moderate in 158 (9.7%), moderately severe in 26 (1.6%), and severe in 8 (0.5%). Most screening‐positive symptoms were in the mild category. Figures 2, 3, 4 present descriptive distributions; they do not establish independent, temporal, or causal associations.

### 3.3 Multivariable Regression Analyses

#### 3.3.1 Depressive‐Symptom Severity

Because the proportional‐odds assumption was not supported, the depression analysis used bias‐reduced multinomial logistic regression, with minimal depressive symptoms as the reference outcome. For mild versus minimal symptoms, higher odds were observed with moderate physical activity (aOR, 1.64; 95% CI, 1.24–2.16), harassment history (aOR, 1.35; 95% CI, 1.03–1.77), peer pressure (aOR, 2.32; 95% CI, 1.83–2.93), 3–4 h of daily screen time (aOR, 1.84; 95% CI, 1.29–2.63), and moderate versus good academic performance (aOR, 1.76; 95% CI, 1.31–2.35). Sleeping 7–8 h (aOR, 0.56; 95% CI, 0.41–0.75), strong social support (aOR, 0.69; 95% CI, 0.53–0.88), and excellent versus good academic performance (aOR, 0.70; 95% CI, 0.51–0.95) were associated with lower odds.

For moderate versus minimal symptoms, second‐year status (aOR, 1.80; 95% CI, 1.22–2.66), harassment history (aOR, 2.39; 95% CI, 1.59–3.60), peer pressure (aOR, 4.07; 95% CI, 2.69–6.18), and moderate versus good academic performance (aOR, 2.13; 95% CI, 1.36–3.34) were associated with higher odds. Sleeping 7–8 h (aOR, 0.22; 95% CI, 0.12–0.38) and strong social support (aOR, 0.42; 95% CI, 0.28–0.64) were associated with lower odds. The complete estimates are presented in Table 1.

**Table 1:** Bias‐reduced multinomial logistic regression for depressive‐symptom severity among early‐year medical students (*N* = 1630).

Predictor comparison	Mild vs. minimal	Moderate vs. minimal	Moderately severe/severe vs. minimal
Female vs. male	1.18 (0.91–1.53)	1.34 (0.84–2.13)	0.31 (0.12–0.82)
Hostel vs. family residence	1.02 (0.81–1.29)	1.22 (0.83–1.79)	2.36 (1.01–5.49)
Second vs. first academic year	1.09 (0.87–1.37)	1.80 (1.22–2.66)	21.49 (5.92–78.04)
Co‐curricular participation: yes vs. no	1.08 (0.84–1.38)	1.27 (0.84–1.91)	0.41 (0.16–1.06)
Physical activity: moderate vs. sedentary	1.64 (1.24–2.16)	1.26 (0.81–1.96)	0.54 (0.22–1.32)
Physical activity: high vs. sedentary	1.12 (0.78–1.61)	1.19 (0.65–2.17)	1.20 (0.29–4.88)
Sleep: 7–8 h/night vs. < 6 h/night	0.56 (0.41–0.75)	0.22 (0.12–0.38)	0.17 (0.05–0.59)
Sleep: irregular vs. < 6 h/night	0.92 (0.69–1.24)	0.84 (0.54–1.29)	0.63 (0.25–1.60)
Family history of mental illness: yes vs. no	1.22 (0.86–1.72)	1.11 (0.66–1.86)	1.32 (0.48–3.67)
Harassment history: yes vs. no	1.35 (1.03–1.77)	2.39 (1.59–3.60)	0.96 (0.39–2.41)
Peer pressure: yes vs. no	2.32 (1.83–2.93)	4.07 (2.69–6.18)	10.79 (3.79–30.73)
Counseling access: yes vs. no	0.85 (0.66–1.10)	0.71 (0.45–1.13)	1.72 (0.71–4.17)
Strong social support: yes vs. no	0.69 (0.53–0.88)	0.42 (0.28–0.64)	0.72 (0.29–1.79)
Family support: yes vs. no	1.31 (0.88–1.96)	1.18 (0.66–2.09)	0.18 (0.07–0.45)
Coping mechanism: positive vs. negative	1.01 (0.75–1.35)	0.78 (0.50–1.21)	0.10 (0.04–0.29)
Screen time: 3‐4 h/day vs. < 2 h/day	1.84 (1.29–2.63)	1.69 (0.85–3.35)	8.89 (0.55–143.35)
Screen time: > 5 h/day vs. < 2 h/day	1.46 (0.99–2.14)	1.42 (0.69–2.90)	12.13 (0.75–196.31)
Academic performance: failing/poor vs. good	0.51 (0.21–1.22)	0.38 (0.08–1.74)	0.15 (0.02–1.03)
Academic performance: moderate vs. good	1.76 (1.31–2.35)	2.13 (1.36–3.34)	1.18 (0.50–2.79)
Academic performance: excellent vs. good	0.70 (0.51–0.95)	0.99 (0.58–1.69)	0.10 (0.01–1.55)

*Note:* Values are aORs (95% Wald CIs) from one bias‐reduced multinomial logistic regression using Jeffreys‐prior penalization. Minimal depressive symptoms are the reference outcome. All listed covariates were entered simultaneously. Descriptive outcome counts were minimal, *n* = 688; mild, *n* = 750; moderate, *n* = 158; moderately severe, *n* = 26; and severe, *n* = 8. Moderately severe and severe categories were combined for multivariable modeling (*n* = 34).

### 3.4 Anxiety‐Symptom Severity

In Table 2 anxiety analysis also used bias‐reduced multinomial logistic regression with minimal anxiety as the reference outcome. For mild versus minimal anxiety, female gender (aOR, 1.63; 95% CI, 1.26–2.11), moderate physical activity (aOR, 1.53; 95% CI, 1.17–2.00), harassment history (aOR, 1.42; 95% CI, 1.10–1.82), peer pressure (aOR, 1.51; 95% CI, 1.20–1.89), 3–4 h of daily screen time (aOR, 1.68; 95% CI, 1.17–2.41), and moderate versus good academic performance (aOR, 1.47; 95% CI, 1.12–1.93) were associated with higher odds. Second‐year status, co‐curricular participation, and sleeping 7–8 h were associated with lower odds.

**Table 2:** Bias‐reduced multinomial logistic regression for anxiety‐symptom severity among early‐year medical students (*N* = 1630).

Predictor comparison	Mild vs. minimal	Moderate vs. minimal	Severe vs. minimal
Female vs. male	1.63 (1.26–2.11)	1.35 (0.77–2.35)	1.15 (0.39–3.43)
Hostel vs. family residence	1.03 (0.82–1.29)	1.17 (0.74–1.87)	2.58 (0.97–6.87)
Second vs. first academic year	0.71 (0.57–0.89)	2.20 (1.35–3.57)	29.84 (3.26–273.21)
Co‐curricular participation: yes vs. no	0.74 (0.58–0.94)	1.15 (0.71–1.87)	2.58 (0.80–8.37)
Physical activity: moderate vs. sedentary	1.53 (1.17–2.00)	1.37 (0.81–2.31)	0.46 (0.15–1.38)
Physical activity: high vs. sedentary	1.23 (0.86–1.76)	0.91 (0.40–2.05)	1.09 (0.26–4.62)
Sleep: 7–8 h/night v.s < 6 h/night	0.69 (0.51–0.92)	0.29 (0.16–0.52)	0.11 (0.02–0.59)
Sleep: irregular vs. < 6 h/night	1.13 (0.85–1.49)	0.54 (0.33–0.91)	0.58 (0.21–1.61)
Family history of mental illness: yes vs. no	1.25 (0.91–1.72)	0.71 (0.37–1.34)	1.29 (0.43–3.84)
Harassment history: yes vs. no	1.42 (1.10–1.82)	1.44 (0.88–2.35)	1.76 (0.60–5.15)
Peer pressure: yes vs. no	1.51 (1.20–1.89)	6.42 (3.59–11.49)	37.23 (3.54–391.86)
Counseling access: yes vs. no	0.95 (0.74–1.22)	1.21 (0.72–2.05)	1.10 (0.40–3.03)
Strong social support: yes vs. no	0.92 (0.72–1.17)	0.45 (0.27–0.75)	1.00 (0.34–2.89)
Family support: yes vs. no	1.29 (0.88–1.89)	0.47 (0.26–0.86)	0.12 (0.04–0.37)
Coping mechanism: positive vs. negative	0.82 (0.63–1.08)	0.93 (0.55–1.56)	0.40 (0.14–1.14)
Screen time: 3–4 h/day vs. < 2 h/day	1.68 (1.17–2.41)	1.06 (0.50–2.24)	4.55 (0.31–66.40)
Screen time: > 5 h/day vs. < 2 h/day	1.41 (0.96–2.08)	0.89 (0.40–1.97)	4.06 (0.28–58.94)
Academic performance: failing/poor vs. good	1.18 (0.51–2.74)	0.18 (0.01–3.55)	0.86 (0.12–6.35)
Academic performance: moderate vs. good	1.47 (1.12–1.93)	1.84 (1.09–3.12)	0.80 (0.27–2.37)
Academic performance: excellent vs. good	0.96 (0.71–1.31)	0.95 (0.48–1.86)	0.10 (0.01–1.38)

*Note:* Values are aORs (95% Wald CIs) from one bias‐reduced multinomial logistic regression using Jeffreys‐prior penalization. Minimal anxiety is the reference outcome. All listed covariates were entered simultaneously. Outcome counts were minimal, *n* = 874; mild, *n* = 642; moderate, *n* = 94; and severe, *n* = 20. Failing and poor academic performance were combined because only five participants reported failing performance. Bold values have 95% CIs excluding 1.00. Severe‐anxiety estimates are imprecise because of the small number of severe cases.

For moderate versus minimal anxiety, second‐year status (aOR, 2.20; 95% CI, 1.35–3.57), peer pressure (aOR, 6.42; 95% CI, 3.59–11.49), and moderate versus good academic performance (aOR, 1.84; 95% CI, 1.09–3.12) were associated with higher odds. Sleeping 7–8 h (aOR, 0.29; 95% CI, 0.16–0.52), irregular sleep versus less than 6 h (aOR, 0.54; 95% CI, 0.33–0.91), strong social support (aOR, 0.45; 95% CI, 0.27–0.75), and family support (aOR, 0.47; 95% CI, 0.26–0.86) were associated with lower odds.

For severe versus minimal anxiety, peer pressure (aOR, 37.23; 95% CI, 3.54–392), second‐year status (aOR, 29.84; 95% CI, 3.26–273), sleeping 7–8 h (aOR, 0.11; 95% CI, 0.02–0.59), and family support (aOR, 0.12; 95% CI, 0.04–0.37) had confidence intervals excluding 1. These estimates should be interpreted cautiously because only 20 participants had severe anxiety and the confidence intervals were very wide.

## 4 Discussion

In this multicenter sample of 1630 first‐ and second‐year medical students, 756 (46.4%) screened positive for anxiety symptoms and 942 (57.8%) for depressive symptoms. Peer pressure showed the most consistent association with greater symptom severity across both outcomes. Harassment, academic stage, sleep, social support, family support, screen time, physical activity, and academic performance were associated with selected outcome contrasts. The findings are cross‐sectional associations from screening instruments, not clinical diagnoses or causal effects.

The symptom prevalence was higher than the pooled estimates reported in international meta‐analyses, but direct comparison is limited by differences in sampling, instruments, cutoffs, time periods, and educational stages [3, 4]. The present study focused on first‐ and second‐year students; results should not be generalized to later preclinical or clinical years, when patient‐care responsibilities, workloads, and available coping resources differ.

Gender associations were outcome‐specific rather than uniform. Female students had higher adjusted odds of mild versus minimal anxiety, but confidence intervals included 1 for moderate and severe anxiety and for mild and moderate depressive symptoms. This pattern illustrates why a single undifferentiated odds ratio would be misleading after the proportional‐odds assumption was rejected. Biological, sociocultural, reporting, and unmeasured contextual explanations remain possible, but the present data cannot distinguish among them.

Peer pressure was associated with progressively larger odds ratios at higher symptom contrasts, particularly moderate and severe anxiety and moderate or higher depressive symptoms. Harassment was associated with mild and moderate depressive symptoms and mild anxiety. These findings are consistent with literature describing interpersonal and academic stress during medical training [5, 6, 7, 8], but the study‐specific binary items did not capture frequency, severity, source, or context. The estimates should therefore be interpreted as broad indicators rather than as precise measures of interpersonal exposure.

Sleep was one of the clearest behavioral correlates. Sleeping 7–8 h, compared with less than 6 h, was associated with lower odds across multiple contrasts. Because symptoms can disrupt sleep and poor sleep can worsen distress, reverse causation and bidirectionality are plausible. Similarly, associations with physical activity, screen time, coping, and academic performance do not establish that changing any single behavior would cause symptom improvement.

Strong social support was associated with lower odds of mild and moderate depressive symptoms and moderate anxiety. Family support was associated with lower odds of moderate and severe anxiety and the upper depressive‐symptom contrast. These findings support further evaluation of confidential peer, family, mentorship, counseling, and referral pathways, while avoiding the assumption that any program is effective in this setting without implementation and outcome evaluation [1, 12, 13].

### 4.1 Service‐Planning Implications

Participating medical colleges could consider evaluating confidential and accessible student‐support pathways, including counseling and referral options, peer or mentorship programs, sleep and stress‐management education, and safe mechanisms for reporting harassment. Implementation should include safeguards, clinical escalation pathways, monitoring of uptake, and assessment of benefits and harms [12, 13]. The present study does not establish effectiveness or cost‐effectiveness.

### 4.2 Financial and Health‐System Implications

Financial burden, service utilization, treatment costs, out‐of‐pocket expenditure, academic interruption, and health‐system demand were not measured. The findings should not be interpreted as evidence that the observed symptoms caused financial or system‐level burden. These questions require studies that directly measure help‐seeking, use of care, costs, educational outcomes, and longer‐term functioning.

### 4.3 Limitations

This study has several limitations. First, its cross‐sectional design precludes assessment of temporality or causality, and several associations may be bidirectional. Second, the PHQ‐9 and GAD‐7 are screening instruments rather than diagnostic interviews. Third, several behavioral and psychosocial predictors were brief study‐specific self‐report items. Nondifferential misclassification could attenuate associations, whereas differential reporting or common‐method bias could shift estimates in either direction. Stigma and social‐desirability concerns may have led to underreporting, while transient distress or heightened symptom awareness may have increased reporting. Fourth, excluding students receiving psychiatric treatment may have reduced the observed symptom burden and limits applicability to students already in care. Fifth, colleges were selected according to accessibility and institutional permission, and the sample was restricted to first‐ and second‐year students in Islamabad; generalizability to later years, other regions, and all Pakistani medical schools is limited. Lastly, severe anxiety (*n* = 20) and moderately severe/severe depressive symptoms (*n* = 34) were uncommon, and sparse cells produced separation. Bias‐reduced models yielded finite estimates, but upper‐severity estimates remain imprecise, and some are highly sensitive to small cell counts. Finally, financial outcomes, service use, treatment costs, academic interruption, and health‐system effects were not measured.

## 5 Conclusion

Anxiety and depressive symptoms were common among first‐ and second‐year medical students at participating colleges in Islamabad. Peer pressure, harassment, sleep, academic year, support, and selected behavioral and academic characteristics were associated with symptom‐severity contrasts. These findings do not establish causality or psychiatric diagnosis. Medical colleges should evaluate confidential, accessible support and referral pathways, while longitudinal and implementation research examines temporality, service use, educational outcomes, and costs.

## Author Contributions

**Farah Rashid:** conceptualization, methodology, investigation, formal analysis, software, data curation, writing – original draft, writing – review and editing. **Iffat Noor:** software, data curation, investigation, resources, writing – original draft. **Wajeha Najeeb:** software, data curation, formal analysis, validation. **Hadiya Rashed Siddiqui:** methodology, software, resources, data curation, investigation. **Hafiz Muhammad Ali:** methodology, investigation, validation, data curation. **Abid Malik:** supervision, writing – review and editing, resources, project administration. **Atif Rahman:** supervision, resources, project administration, writing – review and editing. All authors contributed to manuscript development, reviewed the final version, and approved it for submission.

## Funding

The authors have nothing to report.

## Disclosure

The corresponding author (Farah Rashid) affirms that this manuscript is an honest, accurate, and transparent account of the study being reported; that no important aspects of the study have been omitted; and that any discrepancies from the study as planned (and, if relevant, registered) have been explained.

## Ethics Statement

All procedures involving human participants were conducted in accordance with institutional ethical standards and the Declaration of Helsinki and its later amendments. Ethical approval was obtained from the Institutional Review Board Health Services Academy (No. 00009 IHSA/P/D‐2022).

## Consent

Participants were informed that mental‐health support was available if the survey caused distress. Written informed consent was obtained from all participants. Confidentiality and anonymity were maintained.

## Conflicts of Interest

The authors declare no conflicts of interest.

## Data Availability

Data analyzed in this study are reported in the article. Any sharing of deidentified data is subject to institutional approvals and applicable data‐governance requirements.
